# The transcription factor DEC1 (stra13, SHARP2) is associated with the hypoxic response and high tumour grade in human breast cancers

**DOI:** 10.1038/sj.bjc.6602059

**Published:** 2004-08-24

**Authors:** J Chakrabarti, H Turley, L Campo, C Han, A L Harris, K C Gatter, S B Fox

**Affiliations:** 1 Nuffield Department of Clinical Laboratory Sciences, John Radcliffe Hospital, Oxford OX3 9DU, UK; 2 Cancer Research UK Molecular Oncology Laboratory, Weatherall Institute of Molecular Medicine, John Radcliffe Hospital, Oxford OX3 9DS, UK

**Keywords:** DEC1, breast, tumours, hypoxia, HIF-1*α*

## Abstract

DEC1, also known as SHARP-2 or Stra13, plays important roles in embryonic development, proliferation, apoptosis and cell differentiation in the mouse. DEC1 was recently identified as hypoxically induced in cDNA microarray studies of the human renal carcinoma cell line RCC4, to be regulated through hypoxia-inducible factor (HIF)-1*α* and via HIF-1*α*, able to block adipocyte differentiation. Nevertheless, its distribution and role in hypoxia and differentiation in human breast cancer are unknown. We therefore examined the pattern and level of expression of DEC1 using immunohistochemistry in whole tissue sections in normal, *in situ* and invasive breast carcinomas, and correlated the level of expression of DEC1 and clinicopathological factors and hypoxic tumour markers in 253 invasive carcinomas on tissue microarrays. We observed an increase in DEC1 expression during progression from normal to *in situ* and invasive carcinoma. Expression was not restricted to the tumour cell element but was also observed in endothelial, fibroblasts and inflammatory cells. There was a significant positive correlation between DEC1 and tumour grade (*P*=0.01), HIF-1*α* (*P*=0.04) and the hypoxically regulated gene angiogenin (*P*<0.0001), but no significant associations were observed with patient age (*P*=0.15), lymph node status (*P*=0.8), tumour size (*P*=0.3), oestrogen receptor (*P*=0.45), epidermal growth factor receptor (*P*=0.27) or Chalkley vessel count (*P*=0.45). There was no difference in relapse-free (*P*=0.84) or overall (*P*=0.78) survival. These findings suggest that DEC1 plays an important role in the progression to invasive breast cancer and that it may provide a mechanism by which hypoxia blocks tumour differentiation, and may contribute to a more aggressive phenotype. Reversing this phenotype may alter the biological behaviour of individual tumours.

Hypoxia is increasingly recognised to play a major role in determining tumour behaviour being associated with an aggressive phenotype and resistance to chemo- and radiotherapeutic interventions. The transcriptional complex hypoxia-inducible factor (HIF) has emerged as a key regulator mediating many cellular responses necessary to adapt to changes in oxygen tension (reviewed in Maxwell *et al*, 2001; Goonewardene *et al*, 2002; Pugh and Ratcliffe, 2003). The HIF complex is composed of a heterodimer of HIF-1*α* or HIF-2*α* and HIF-*β* (also known as aryl-hydrocarbon nuclear translocator). Hypoxia-inducible factor-*β* is constitutively expressed and is involved in several transcriptional systems, whereas the two HIF-*α* subunits are specific to the hypoxic pathway. In normoxic conditions, the HIF-*α* units are unstable since two prolyl residues within the oxygen-dependent degradation domains of HIF-*α* subunits are hydroxylated by prolyl hydroxylases and dioxygen as a co-substrate (reviewed in Pugh and Ratcliffe, 2003). This results in one oxygen being incorporated into the prolyl residue of HIF-*α*, allowing rapid targeting and degradation by the proteasome pathway via the von Hippel–Lindau (VHL) protein and the ubiquitin E3 ligase complex. However, in hypoxia, as frequently occurs within tumours, there is insufficient oxygen to allow this process resulting in HIF-*α* stabilisation and translocation to the nucleus, where it is able to bind HIF-*β*. The complex then recruits co-activators that bind specific DNA hypoxia response elements (HREs), resulting in increased mRNA transcription.

Many HIF target genes are beneficial to tumour, including those involved in iron metabolism (e.g. transferrin), angiogenesis (e.g. vascular endothelial growth factor), glucose metabolism (glucose transporters), proliferation (insulin growth factor II), endothelial adhesion and pH regulation (carbonic anhydrase IX) (Zund *et al*, 1996; Semenza, 2000; Wenger, 2000). Recently, using a cDNA microarray in the renal carcinoma cell line RCC4, which was either defective or competent for VHL, we identified DEC1 (differentially expressed in chondrocytes) to be another such hypoxia-inducible gene (Wykoff *et al*, 2000b).

DEC1 was originally identified independently by three groups studying different mammalian systems of differentiation (Boudjelal *et al*, 1997; Rossner *et al*, 1997; Shen *et al*, 1997). DEC1 (also known as split and hairy related protein (SHARP) 2 or stimulated with retinoic acid (Stra) 13) is a 412 amino-acid transcription factor whose RNA is expressed in most embryonic and adult tissues. DEC1 shows high amino-acid sequence similarity to the *Drosophila* hairy and enhancer of Split m7 and mammalian HES1 transcription factors across its basic helix–loop–helix (bHLH) domain, but it lacks the C-terminal WRPW domain that accounts for their transcriptional repressive functions, indicating that DEC1 is a member of a distinct bHLH subfamily. DEC1 binds to DNA, demonstrates transcriptional repressive activity and interacts with several subunits of RNA polymerase II, suggesting that DEC1 represses transcription through modulation of the basal transcriptional machinery and histone deacetylase (Boudjelal *et al*, 1997; Sun and Taneja, 2000).

DEC1 is reported to have roles in proliferation (Boudjelal *et al*, 1997), apoptosis (Li *et al*, 2002) and cell differentiation (Fisher *et al*, 1996). Indeed, it is possible that DEC1 functions as a HIF effector to mediate the effect of hypoxia on differentiation, since oxygen tension alters differentiation of cytotrophoblast, megakaryocytes, osteochondrocytes, bone marrow cells, adipocytes and neurons (Caniggia *et al*, 2000; Mostafa *et al*, 2000; Studer *et al*, 2000; Lennon *et al*, 2001; Yun *et al*, 2002). Nevertheless, since little is known about DEC1 function in human physiological and pathological processes, we raised a polyclonal antibody to investigate the expression profile of DEC1 in normal and neoplastic tissues by immunohistochemistry to help determine its role. This has shown DEC1 to be widely expressed in the nuclei of cells in many tissues, but with a restricted pattern of expression. Although others and we have also reported the expression of DEC1 in several tumour types (Ivanova *et al*, 2001; Li *et al*, 2002, 2003; Giatromanolaki *et al*, 2003), the pattern and level of expression of DEC1 in breast tissues and tumours have not been systematically investigated and the potential role in this tumour type is unknown. Thus, in order to characterise further the significance of DEC1 in normal and neoplastic breast tissues, we have investigated the distribution and level of expression of DEC1 and correlated this with clinicopathological and hypoxic tumour markers in a large series of breast carcinomas.

## MATERIALS AND METHODS

### Patients and tumours

Whole tissue sections from 15 pure *in situ* breast carcinomas (five low, five intermediate and five high nuclear grade), 101 invasive breast carcinomas and 14 histologically normal breast tissues derived from reduction mammoplasties, together with microarrayed tumour cores from 253 breast carcinomas, were collected from patients undergoing surgery at the John Radcliffe Hospital, Oxford, UK. Tumours were treated by mastectomy (*n*=58) or lumpectomy (*n*=195), axillary node sampling with node status confirmed histologically. Primary histological types where known included 188 ductal carcinomas (not otherwise specified), 18 lobular and 15 others. Grading was performed according to the modified Bloom and Richardson method. The clinicopathological characteristics of the series are presented in Table 1

**Table 1:** Correlation analyses between DEC1 and clinicopathological, angiogenic and hypoxia markers for 253 invasive breast carcinomas studied by tissue microarray

	**DEC1 negative**	**DEC1 positive**	***P*-value**
No. of patients	68	185	

*Age*			
<50	15	58	0.15
⩾50	53	127	
			
*Nodal status*			
Neg	37	104	0.80
Pos	31	81	
			
*Tumour size (cm)*			
⩽2 cm	41	98	0.30
>2 cm	27	87	
			
*Grade*			
I	19	26	0.01^*^
II	16	56	
III	12	56	
			
*ER*			
Neg	19	61	0.45
Pos	49	124	
			
*EGFR*			
Neg	30	67	0.27
Pos	37	114	
			
*Chalkley count*			
Low	3	29	0.45
High	5	23	
			
*Angiogenin*			
Neg	46	76	0.0001^*^
Pos	19	101	
			
*HIF-1α*			
Neg	54	124	0.04^*^
Pos	8	42	

ER=oestrogen receptor; EGFR=epidermal growth factor receptor; HIF=hypoxia-inducible factor. ^*^Denotes significance, where *n*<253 data are unavailable.

. In patients <50 years, adjuvant cyclophosphamide, methotrexate and 5-fluorouracil (CMF) was administered if tumours were node positive, or oestrogen receptor (ER) negative and/or ⩾3 cm. Patients ⩾50 years with ER-negative, node-positive tumours also received CMF. The median follow-up was 7.3 (range 0.4–11) years, in which there were 89 relapses and 62 deaths.

### DEC1 immunohistochemistry

Formalin-fixed paraffin-embedded sections (4 *μ*m) of normal and primary breast tumours were immunostained with the rabbit polyclonal antibody CW27 as described previously, using the EnVision^TM^ Detection Kit, Peroxidase/DAB (DAKO, Denmark) (Giatromanolaki *et al*, 2003). The pattern of DEC1 expression was determined from whole tissue sections, whereas the level of nuclear expression was derived using tissue microarrays. One core from the tumour periphery, which is reported to be most biologically relevant (Weidner *et al*, 1991), and the following scoring system were used: negative=0, weak nuclear staining=1, moderate nuclear staining=2 or strong nuclear staining=3. Score 2 and 3 tumours were considered positive for DEC1 in statistical analyses. Two observers assessed the localisation and degree of cellular staining using a conference microscope.

### Assessment of HIF-1*α*, angiogenin and vascularity

The anti-HIF-1*α* protein monoclonal antibody ESEE 122 (Talks *et al*, 2000) and rabbit polyclonal antibody against angiogenin (ANG; Santa Cruz Biotechnology; catalogue number sc-9044)(Hartmann *et al*, 1999; Hisai *et al*, 2003) were applied to sections at dilutions of 1 : 40 and 1 : 100, respectively, followed by the Envision-HRP kit (DAKO, Glostrup, Denmark). Evaluation of HIF-1*α* was based on the intensity and extent of nuclear and cytoplasmic reactivity as reported previously (Talks *et al*, 2000), and angiogenin was scored with the same scoring system as for DEC1. Tumour vascularity was counted by scanning at low power (× 40–100) for the three areas of highest vascularity before using a 25-point Chalkley point eyepiece graticule at × 250 (0.155 mm^2^) over these hot spots. The graticule was orientated so that the maximum number of points was on or within areas of highlighted vessels. The mean of three graticule points for each tumour was used in the statistical analysis, with the upper third used as a cut point for categorical analysis as determined previously (Fox *et al*, 1995).

## RESULTS

### DEC1 expression in normal, *in situ* and invasive breast carcinomas

#### Whole tissue sections

**Figure 1:**
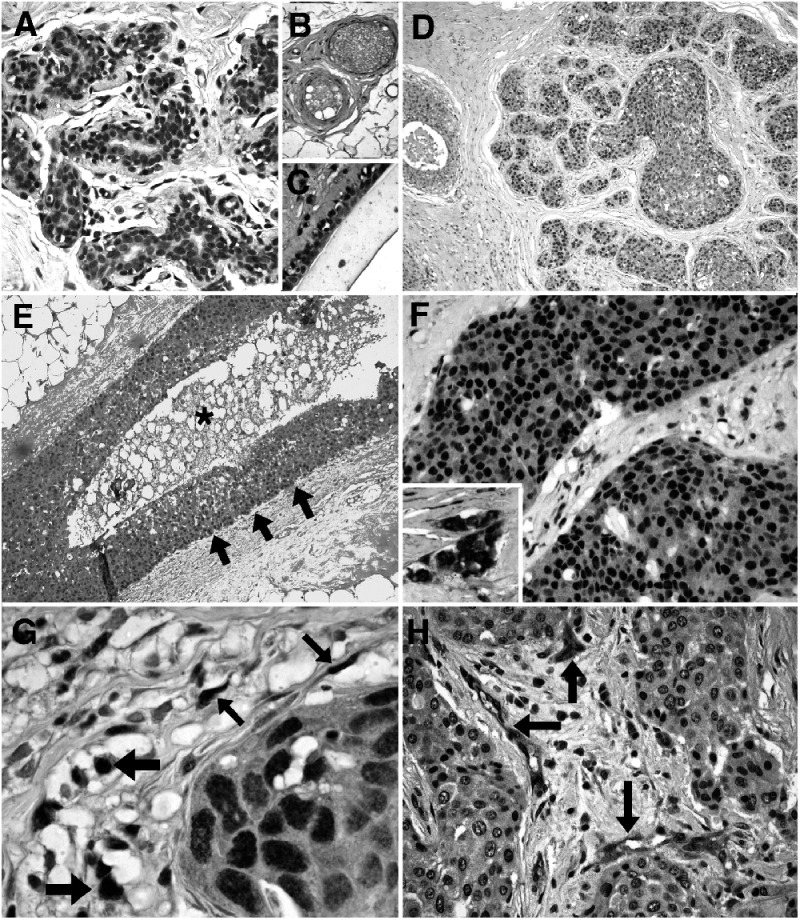
DEC1 expression in normal and neoplastic breast tissues. Patchy and weak predominantly nuclear DEC1 expression in luminal epithelial cells of normal acini (**A**). DEC1 in endothelial cells and fibroblasts around vessels in normal breast (**B**). Myoepithelial and luminal DEC in a normal large duct, together with stromal fibroblast positivity (**C**). Intermediate nuclear grade ductal carcinoma *in situ* (DCIS) of predominant solid pattern, showing moderate DEC1 expression that varies within and between involved ducts (**D**). DEC1 in high nuclear-grade DCIS of comedo type, showing gradual enhancement of expression towards the periphery of the duct and not immediately adjacent to the necrotic area (asterisk) (**E**). Strong nuclear and weak cytoplasmic DEC1 expression in an invasive ductal carcinoma; the inset demonstrates strong nuclear and cytoplasmic staining in a different invasive ductal carcinoma (**F**). Fibroblast (thin arrows) and macrophage (thick arrows) DEC1 staining adjacent to an island of invasive ductal carcinoma (**G**). Strong DEC1 endothelial cell positivity in tumour-associated vessels in an invasive ductal carcinoma showing weak DEC immunopositivity (**H**).

DEC1 expression in normal human breast tissues was weak and patchy, being mostly present in the nuclei and occasionally in the cytoplasm of the epithelial elements. Expression was present predominantly in the luminal epithelial cells of the acini of terminal duct lobular units and in larger ducts (Figure 1A and B

), but myoepithelial cell staining was occasionally present, which was enhanced in areas demonstrating myoid metaplasia. The intensity of DEC1 expression was also increased in areas of apocrine and columnar cell metaplasia. Weak DEC1 expression was also observed in stromal cells of the interlobular and intralobular stroma and in endothelial cells of capillaries (Figure 1C). In *in situ* carcinomas, expression was generally stronger both in the nucleus and the cytoplasm of the neoplastic cells (Figure 1D). One of 15 cases was negative, four cases showed weak staining, five cases moderate staining and five showed strong staining. Expression of DEC1 was variable, both within and between individually affected ducts (Figure 1D). No enhancement was observed adjacent to areas of necrosis and indeed an accentuated expression was observed at the periphery of involved ducts, increasing in the cell layers away from the edge of the necrosis and not immediately adjacent to it (Figure 1E). In the 101 invasive carcinomas, expression of DEC1 was again nuclear but also cytoplasmic in 41 cases, and immunopositivity was stronger and more homogenous throughout the tumour than in normal or *in situ* carcinomas (Figure 1F). Two tumours were negative, 40 showed weak staining, 24 moderate and 35 strong staining. Other tumour elements were also DEC1 positive, including fibroblasts, macrophages and endothelial cells (Figure 1G and H) in 86 out of 101 (85%), 43 out of 101 (42%) and 79 out of 101 (78%) cases, respectively. Although not graded due to the significant variability in the quantity of these elements, expression was usually stronger than that observed in normal tissues.

#### Tissue microarrays

One (<1%) of 253 tumours was negative, 67 (26%) cases showed weak staining, 75 (30%) cases showed moderate staining and 110 (44%) showed strong staining. In all, 68 were considered negative and 185 positive for the statistical analysis. For cytoplasmic staining, 123 (49%) tumours were positive and 130 (51%) were negative.

### Relationship between DEC1 expression, clinicopathological and hypoxic variables and survival

**Figure 2:**
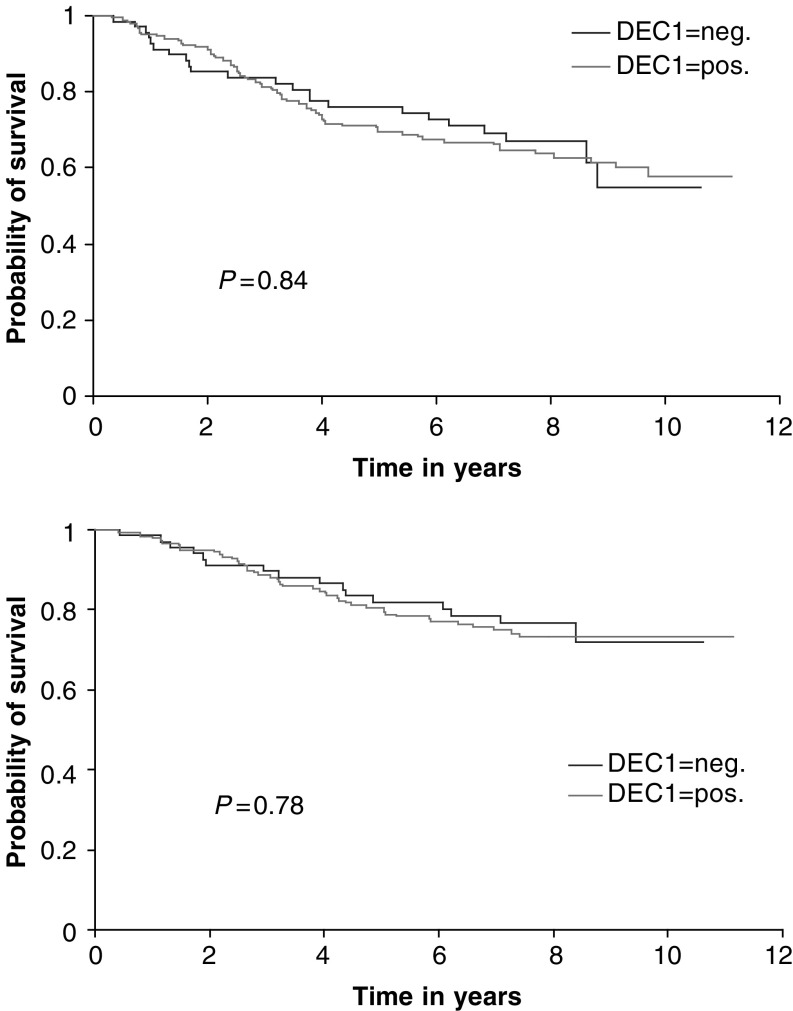
Kaplan and Meier relapse-free survival (upper graph) and overall survival curves (lower graph) stratified by DEC1 expression.

There was a significant positive correlation in 253 invasive carcinomas from tissue microarrays between DEC1 and tumour grade (*P*=0.01), HIF-1*α* (*P*=0.04) and the hypoxically regulated gene (Hartmann *et al*, 1999) angiogenin (*P*<0.0001). No significant associations were observed with patient age (*P*=0.15), lymph node status (*P*=0.8), tumour size (*P*=0.3), ER (*P*=0.45), epidermal growth factor receptor (*P*=0.27) or Chalkley vessel count (*P*=0.45) (Table 1). There was no difference in relapse-free (*P*=0.84) or overall (*P*=0.78) survival in a univariate analysis of patients when tumours were stratified by DEC1 expression (Figure 2

).

## DISCUSSION

DEC1 is a new and structurally different class of bHLH protein. Although little is known about its function in human physiological and pathological processes, in the mouse DEC1 represses adipocyte, mesodermal and endodermal differentiation but promotes neuronal differentiation (Boudjelal *et al*, 1997; Yun *et al*, 2002). It may also play a role in regulating peripheral T-cell tolerance through elimination of activated lymphocytes (Sun *et al*, 2001), affect cell proliferation (Sun and Taneja, 2000) and apoptosis (Li *et al*, 2002). We and others have identified DEC1 as a VHL target gene (Ivanov *et al*, 1998; Wykoff *et al*, 2000b). In view of the potential of DEC1 to regulate the cellular responses to hypoxia in neoplasia through the above processes, we have examined the pattern and level of expression of DEC1 in normal and neoplastic human breast tissues. In normal breast, we observed only a low level and patchy expression of DEC1, suggesting that it is unlikely to play a role in normal cyclical physiological changes. However, there was increased expression of DEC1 in metaplastic changes, in keeping with its role in cellular differentiation (Shapland *et al*, 1988; Fisher *et al*, 1996).

The incremental increase in DEC1 expression in both the nuclear and cytoplasmic neoplastic cell compartments during progression of normal to *in situ* and invasive carcinoma suggests that DEC1 plays a role in breast cancer progression. It may additionally have a role in stroma since expression was frequently observed particularly in fibroblasts and endothelium. However, the mechanism of DEC1 regulation in tumour cells must be VHL independent since, unlike conventional clear-cell renal cell carcinomas, breast cancers have an intact VHL gene. This is emphasised by the strong DEC1 staining of other tumour elements, including endothelial cells that are genotypically normal. Although little is known about the regulation of DEC1, prostaglandin E2 and transforming growth factor (TGF)-*β* have been reported to induce DEC1, TGF-*β* being frequently expressed in breast carcinomas (Zawel *et al*, 2002; Bek *et al*, 2003).

Since Stra13 is associated with growth arrest, the increase in DEC1 expression in tumours may seem paradoxical (Boudjelal *et al*, 1997; Sun and Taneja, 2000). However, the level of expression alters the effect, with medium levels resulting in an increased proliferation rate of approximately two- to three-fold, with the higher-expressing clones not being viable (Boudjelal *et al*, 1997; Sun and Taneja, 2000).

DEC1 is induced by hypoxia through a HRE (Miyazaki *et al*, 2002), and in our study we have demonstrated a significant association with HIF-1*α* and the hypoxia-induced gene angiogenin (Hartmann *et al*, 1999), supporting its induction by this microenvironmental stress in breast cancer. Nevertheless, unlike CAIX (Wykoff *et al*, 2000a), we did not observe upregulation of DEC1 immediately adjacent to areas of necrosis in either *in situ* or invasive carcinomas, similar to findings obtained by mRNA *in situ* hybridisation studies (Peter Watson, personal communication). Indeed, DEC1 was increasingly expressed in cell layers distant from the edge of the necrotic zone. This pattern of expression overlaps with that of CAIX and HIF-1*α*, with HIF-1*α* located throughout tumours, showing that there is a differential pattern of localisation of hypoxia-associated molecules. The reasons for this are not clear, but may reflect the different half-lives of the proteins and/or regulation by other factors such as TGF-*β*, the latter of which is associated with breast cancer progression (Gorsch *et al*, 1992; Zawel *et al*, 2002).

Nevertheless, the marked induction of this differentiation factor by hypoxia within tumours suggests that it may be predominantly regulated by hypoxia and that hypoxia may have a role in differentiation. Indeed, a positive association between tumour grade and HIF-1*α* has been reported. Thus, the significant positive correlation between high DEC1 expression and high tumour grade that we describe, an association that has been reported in lung cancers (Giatromanolaki *et al*, 2003) is in accordance with its induction by HIF (Bos *et al*, 2001) and Stra13's ability to repress differentiation in embryogenesis and adipocytes (Boudjelal *et al*, 1997; Yun *et al*, 2002). The mechanism of this repression may be through interaction with other transcription factors such as TFB, TFIIB and USF, since DEC1, unlike other family members, does not bind to E box, N box or C box sequences (Yun *et al*, 2002). DEC1 is also reported to antagonise serum deprivation-induced apoptosis through selective repression of pro-caspases 3, 7 and 9, but not 8, suggesting the blockage of mitochondrial apoptotic pathways (Li *et al*, 2002). Thus, DEC1 may play a further role in rescuing high-grade tumours from widespread programmed cell death (Gandhi *et al*, 1998).

Although our findings suggest that DEC1 may contribute to mediating the aggressive phenotype of hypoxic tumours, we did not demonstrate a significant association between DEC1-positive tumours and relapse-free or overall survival that has been reported for hypoxic node-negative and node-positive breast tumours (Schindl *et al*, 2002; Bos *et al*, 2003). This is likely to be due to the accumulated effects of the many classes of genes that the HIF response activates. Nevertheless, data derived from mouse podocytes suggest that DEC1 may play an important cytoprotective role against reactive oxygen species through upregulating haeme oxygenase-1 and decreasing NADPH oxidase (Bek *et al*, 2003). Thus, the level of DEC1 in tumours should be assessed for a relation in response to radiotherapy.

Recently, DEC2, a human DEC1 homologue, has been cloned and has also been shown to be hypoxically induced (Fujimoto *et al*, 2001; Miyazaki *et al*, 2002). Data to date suggest that, whereas DEC1 is mostly expressed in cancer, DEC2 expression is higher in the adjacent normal tissues. Indeed, forced expression of DEC1 resulted in repression in the activity of a DEC2 promoter reporter (Azmi *et al*, 2003). It will thus be of interest to establish the role of this protein in the hypoxic pathway.

In summary, we have shown that DEC1 expression increases on progression from normal to *in situ* and invasive carcinoma, supporting a significant role for this transcription factor in breast neoplasia. We have further shown that DEC1 expression is strongly associated with HIF-1*α*, the hypoxically induced protein angiogenin and tumour grade, suggesting a role for DEC1 in blocking tumour differentiation and potentially apoptosis. These findings provide mechanisms by which breast tumours in a hypoxic environment acquire a more aggressive phenotype (Schindl *et al*, 2002).
